# Supplementary material for: SENP2-based N-terminal truncation of α-synuclein in Lewy pathology propagation
Source: iScience. 2025 Jan 31;28(2):111935. doi: 10.1016/j.isci.2025.111935 (PMC11869972; doi:10.1016/j.isci.2025.111935)
Supplement: Document S1. Figures S1–S6 [file mmc1.pdf]

iScience, Volume *28*

Supplemental information

SENP2-based N-terminal truncation of α-synuclein in Lewy pathology propagation

Katsutoshi Taguchi, Yoshihisa Watanabe, and Masaki Tanaka

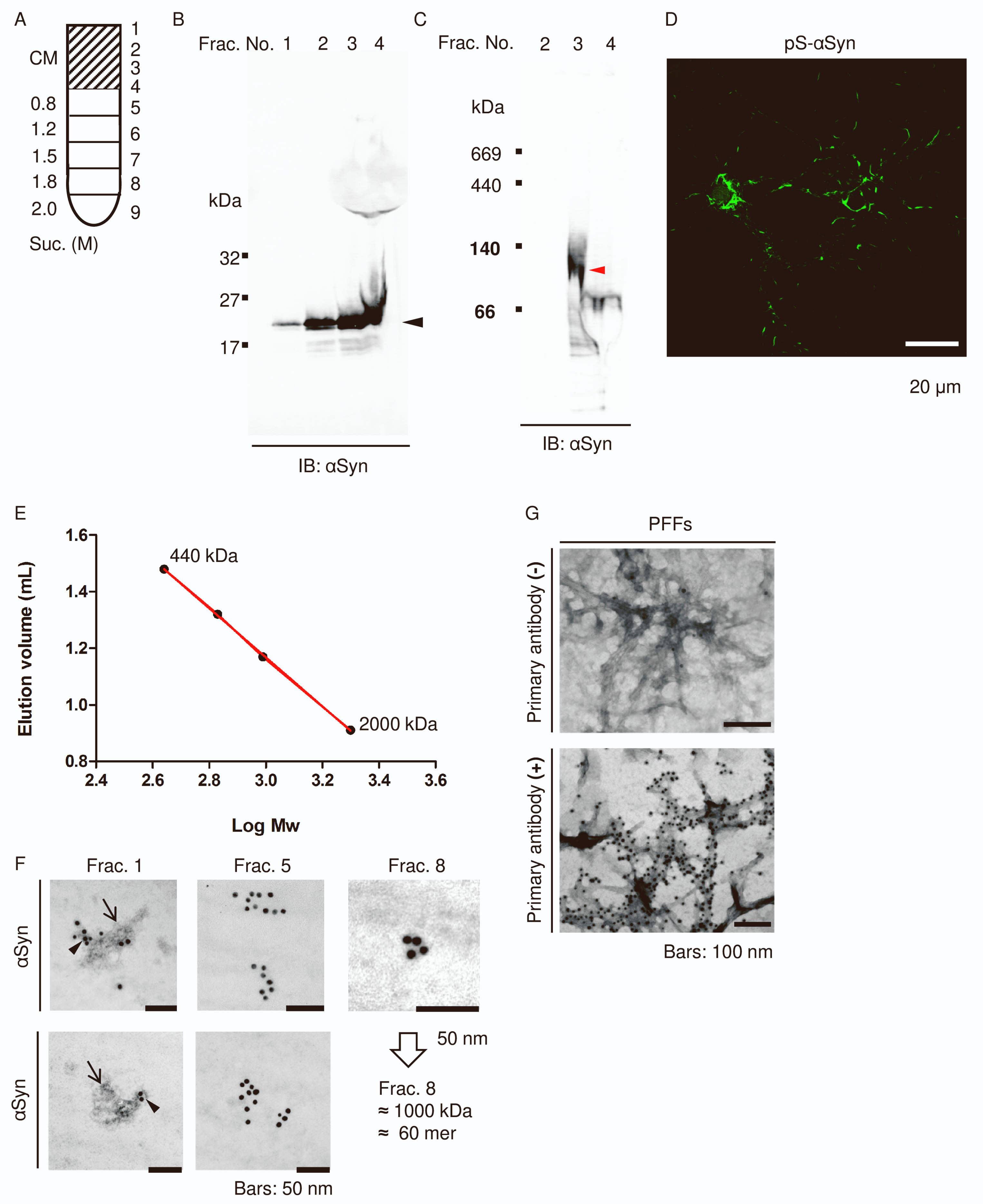

A
CM
0.8
1.2
1.5
1.8
2.0
Suc. (M)
1
2
3
4
5
6
7
8
9
B
Frac. No. 1 2 3 4
kDa
32
27
17
IB: αSyn
C
Frac. No. 2 3 4
kDa
669
440
140
66
IB: αSyn
D
pS-αSyn
20 μm
E
Elution volume (mL)
1.6
1.4
1.2
1.0
0.8
2.4
2.6
2.8
3.0
3.2
3.4
3.6
Log Mw
440 kDa
2000 kDa
F
Frac. 1
Frac. 5
Frac. 8
αSyn
αSyn
50 nm
Frac. 8
≈ 1000 kDa
≈ 60 mer
Bars: 50 nm
G
PFFs
Primary antibody (-)
Primary antibody (+)
Bars: 100 nm

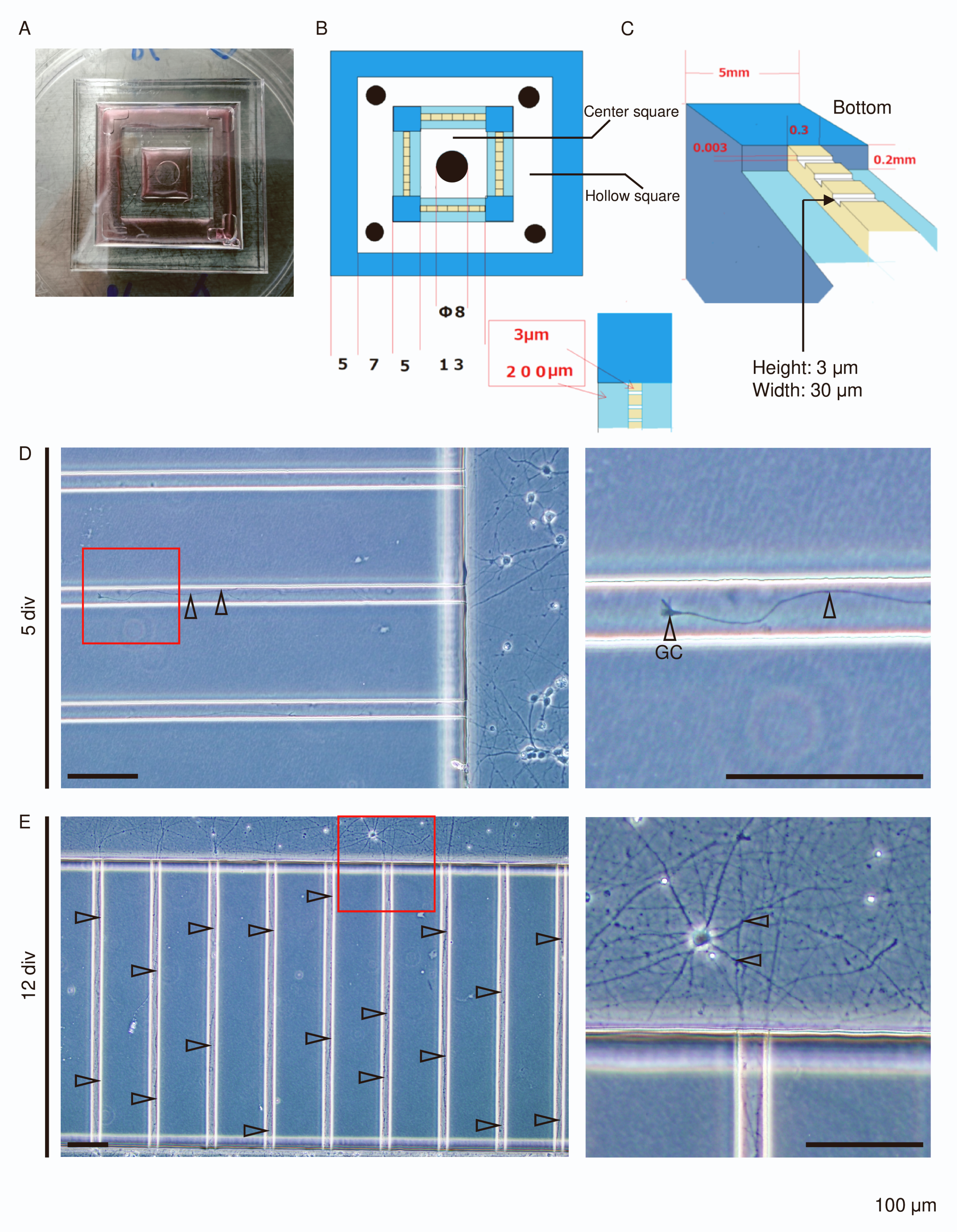
A
B
C
Center square
Hollow square
Φ8
5
7
5
1 3
3μm
2 0 0μm
5mm
Bottom
0.3
0.003
0.2mm
Height: 3 μm
Width: 30 μm
D
5 div
GC
E
12 div
100 μm

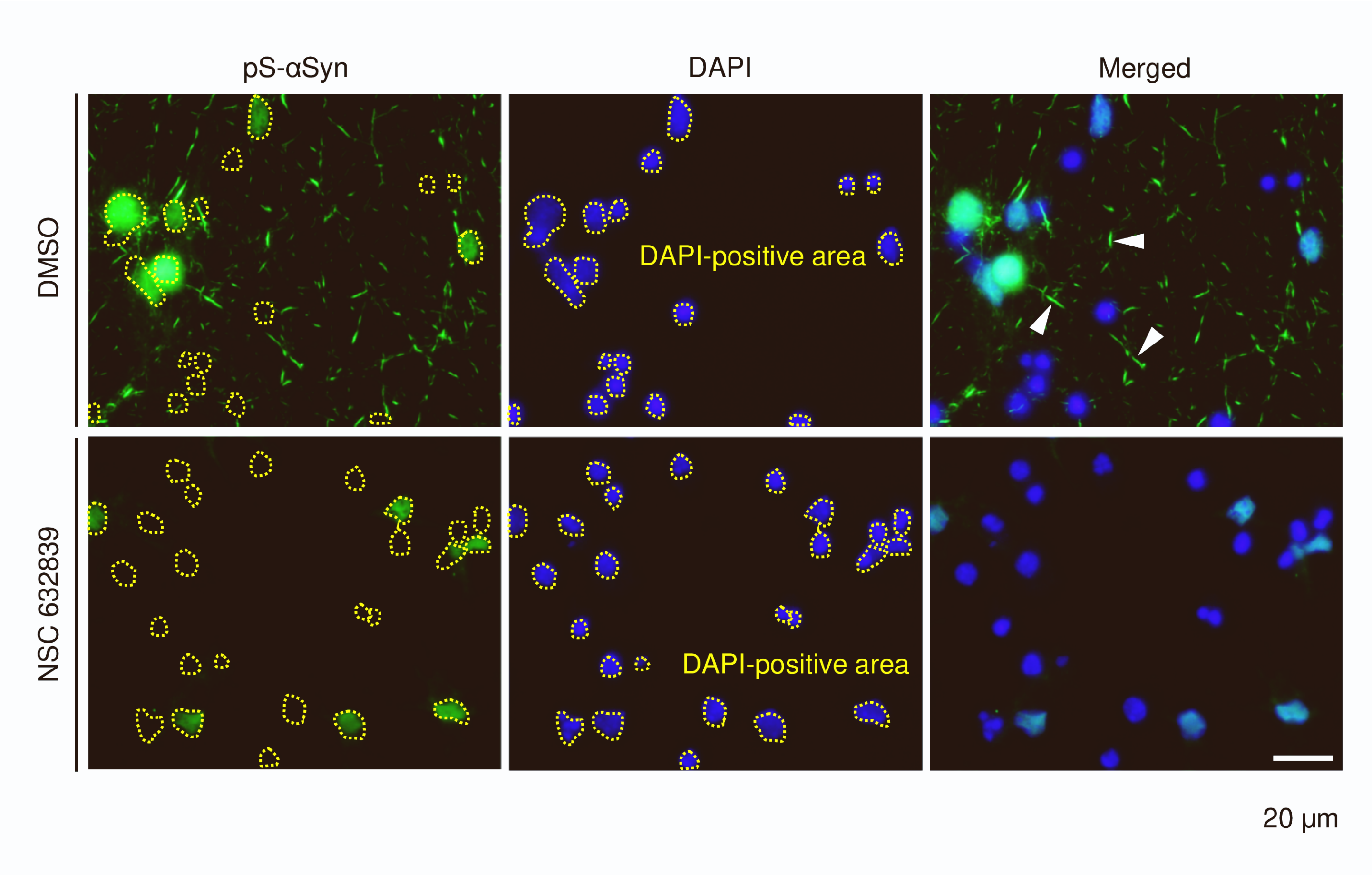
pS-αSyn
DAPI
Merged
DMSO
NSC 632839
DAPI-positive area
DAPI-positive area
20 µm

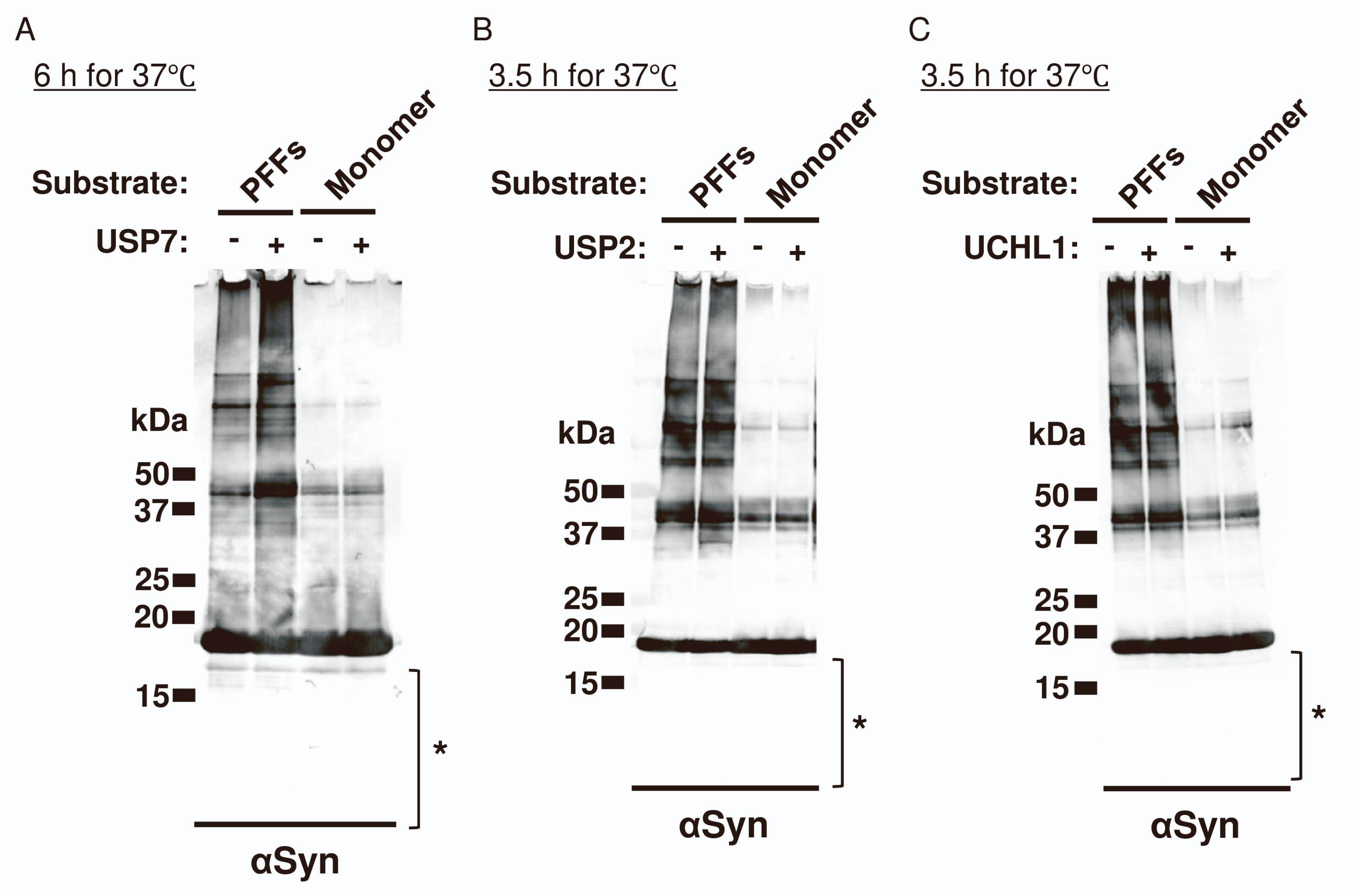
A
6 h for 37℃
Substrate:
PFFs
Monomer
USP7:
- + - +
kDa
50
37
25
20
15
*
αSyn
B
3.5 h for 37℃
Substrate:
PFFs
Monomer
USP2:
- + - +
kDa
50
37
25
20
15
*
αSyn
C
3.5 h for 37℃
Substrate:
PFFs
Monomer
UCHL1:
- + - +
kDa
50
37
25
20
15
*
αSyn

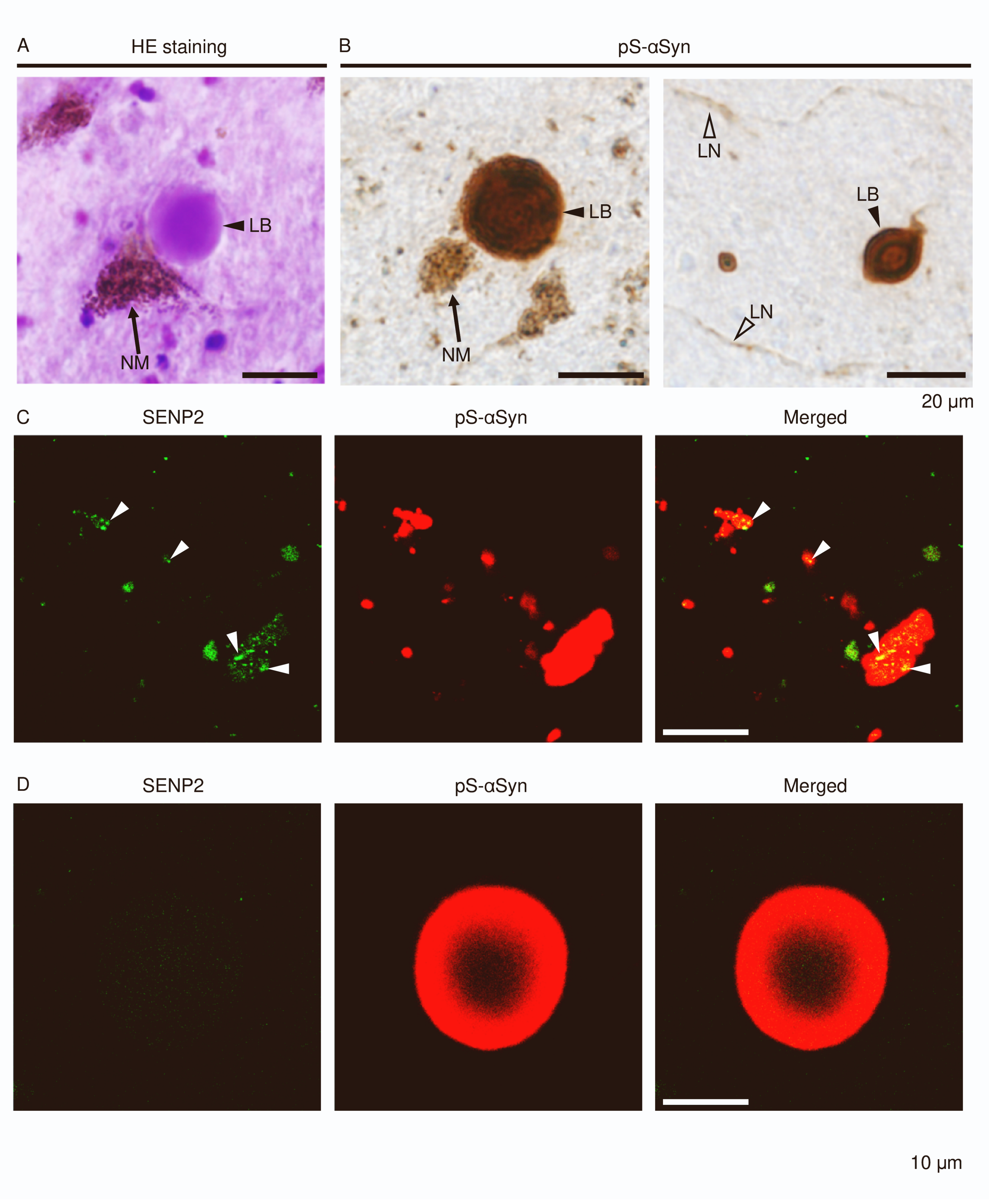

A
HE staining
LB
NM
B
pS-αSyn
LB
NM
LN
LB
LN
20 μm
C
SENP2
pS-αSyn
Merged
D
SENP2
pS-αSyn
Merged
10 μm

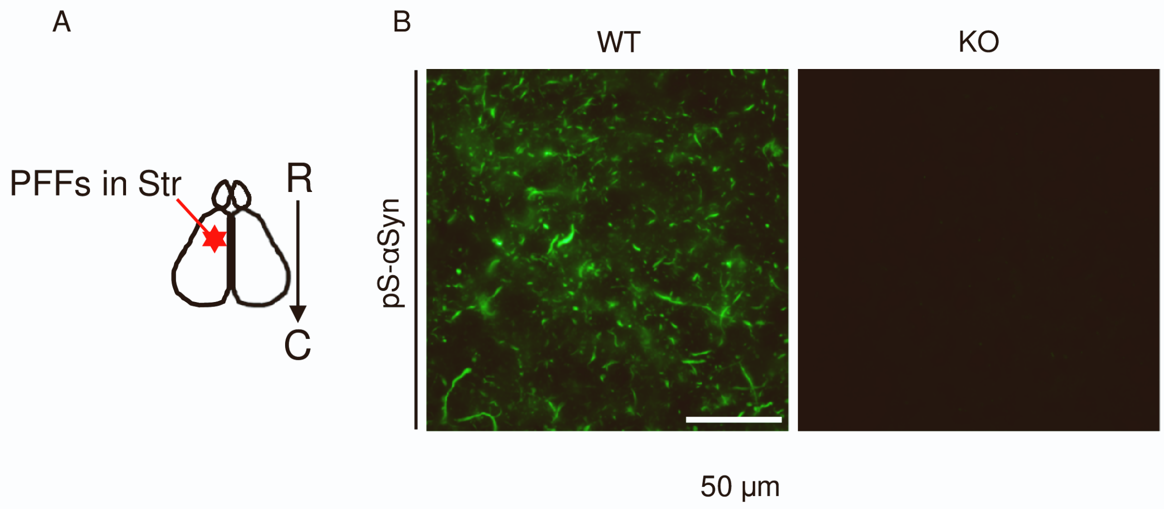
A
B
WT
KO
PFFs in Str
R
C
pS-αSyn
50 μm

Supplemental figure legends

Figure S1. Isolation and characterization of pathological seeds using the batch method, Related to Figure 1. (A) Schematic diagram of fractionation by sucrose-density gradient ultracentrifugation of the conditioned culture medium derived from pathological primary neurons harbouring Lewy body- and Lewy neurite-like aggregates. CM, culture medium. **(B)** SDS-PAGE and an immunoblotting assay of the fractions using anti-αSyn antibody. The numbers indicate the recovered fractions shown in **(A)**. **(C)** Native-PAGE and immunoblotting assay of the fractions. **(D)** The seeding ability of the pathological seeds recovered in Fraction 3. pS-αSyn, phosphorylated αSyn. **(E)** Standard curve for HPLC (Log. molecular weight vs. elution volume). **(F)** Immunoelectron microscopic observation of the αSyn-oligomer species recovered in each HPLC-prepared fraction. **(G)** Immunoelectron microscopic observation of PFFs as a control for αSyn immunolabeling. PFFs, preformed fibrils. Scale bar: 20 μm in **(D)**, 50 nm in **(F)**, and 100 nm in **(G)**.

Figure S2. Development of the microfluidic device for large-scale preparation of pathological seeds, Related to Figure 2. (A–C) Overview of microfluidic device for large-scale preparation of seeds. **(D, E)** Primary neuronal culture using the large-scale microfluidic device for 5 days **(D)** and 12 days **(E)** *in vitro*. Arrowheads show the

extending axons. The region marked by a red square in **(D)** and **(E)** is magnified in each right panel, respectively. GC, growth cone. Scale bar: 100 μm.

Figure S3. Image analysis of the inhibitory effect of NSC632839 on aggregate formation, Related to Figure 3. Phosphorylated-αSyn immunoreactive area, excluding the DAPI-positive area surrounded by dotted lines, was quantitatively analysed because the monoclonal antibody against phosphorylated αSyn #64 tends to react with the nuclei. Arrowheads indicate examples of phosphorylated αSyn-positive aggregates. Scale bar: 20 μm.

Figure S4. Screening assay for possible αSyn-processing enzymes, Related to Figure 3. (A–C) αSyn-processing ability of possible NSC632839-inhibited enzymes was examined by a protease assay using recombinant proteins. As indicated by asterisks, cleaved fragments were not detected in the presence of USP-7 **(A)**, USP-2 **(B)**, or UCHL1 **(C)**. Both PFFs and a monomeric form of αSyn were not processed as appropriate substrates by these enzymes. The reaction time and temperature are indicated in each figure panel.

Figure S5. Presence of SENP2 in Lewy pathology, Related to Figure 4. (A, B) Lewy body, Lewy neurites, and neuromelanin observed in the substantia nigra pars compacta of the PD-brain. **(C, D)** Presence of endogenous SENP2 in amorphous aggregates **(C)**, but not in a typical Lewy body **(D)**. Arrowheads indicate the presence of both SENP2 and phosphorylated αSyn. LB, Lewy body; LN, Lewy neurites; NM, neuromelanin. Scale bar: 20 μm in **(A, B)**, and 10 μm in **(C, D)**.

Figure S6. Endogenous αSyn expression is indispensable for aggregate formation, Related to Figure 5. (A) Schematic diagram of PFF injection into the striatum of the mouse brain. C, caudal; R, rostral; Str, striatum. **(B)** Intense formation of phosphorylated-αSyn aggregates was observed in the WT, but not αSyn-KO, mouse brain. WT, wildtype; KO, knockout. Scale bar: 50 μm.
